# Characterization of human tear proteome reveals differentially abundance proteins in thyroid-associated ophthalmopathy

**DOI:** 10.7717/peerj.13701

**Published:** 2022-07-12

**Authors:** Xiaoqing Zhou, Ruili Wei, Rui Wang

**Affiliations:** 1 Department of Ophthalmology, Shanghai Changzheng Hospital, Shanghai, Shanghai, China; 2 Proteome Research Cente, Shanghai Applied Protein Technology, Shanghai, Shanghai, China

**Keywords:** Hyroid-associated ophthalmopathy, Tears, Label-free LC-MS/MS, Proteomics analysis, Complement and coagulation cascades

## Abstract

**Background:**

Thyroid-associated ophthalmopathy (TAO) is a common orbital inflammatory disease, but the abnormal expression of proteins in tears of TAO patients has not been systematically studied. The purpose of this study is to compare and analyze the total tear protein profile of TAO patients and to provide protein cues for TAO pathogenesis.

**Methods:**

Tear samples were isolated from 30 TAO patients with obvious ocular surface damage and 30 healthy control subjects. Tear samples from 30 individuals were mixed and divided into three sample pools. Easy nano-scale LC-MS/MS based on labeling-free quantitative technology was utilized to profile tear proteome.

**Results:**

Here, electrospray ionization mass spectra and SDS-PAGE results confirmed the good parallelisms among samples. A total of 313 proteins were obtained from six tear pools, among them, 103 differential abundance proteins (DAPs) were identified, including 99 up-regulated DAPs (including APOA1, HV103, IGH, and Transferrin variant) and four down-regulated DAPs (including FABA, VCC1, NUCB2, and E-cadherin) in the TAO group compared with the control group. GO analysis showed that up-regulated DAPs were mainly enriched in lipid metabolism and platelet molecular function, and down-regulated DAPs were involved in binding, cell junction, and cellular process. KEGG results indicated that DAPs were involved in 117 kinds of signal transduction pathways, among which the immune-related pathway of complement and coagulation cascades had the greatest relevance.

**Conclusion:**

In conclusion, label-free LC-MS/MS is an effective strategy for profiling tear proteins component. Our study provides proteins and pathways altered in TAO and provides protein cues for further study on the precise mechanism of TAO pathogenesis.

## Introduction

Thyroid-associated ophthalmopathy (TAO) is an ocular condition characterized by thyroid dysfunction and is a common orbital inflammatory disease. Patients with TAO usually manifest with eyelid retraction, orbital edema, lagophthalmos, proptosis, dysmotility, diplopia, lacrimation, as well as ocular surface irritation (Gupta, Sadeghi & Akpek, 2009). Ocular surface disease is frequently diagnosed in TAO patients especially for dry eye syndrome. For example, the study reported that 39 of 170 (23%) patients with diagnosed autoimmune thyroid diseases match the criteria for keratoconjunctivitis sicca (Coll et al., 1997). The estimated prevalence of TAO in the United States of America was 0.3%, and in the United Kingdom in women was 2.7% and in men was 0.3% (Sahli & Gunduz, 2017). In addition, TAO usually exhibits clinical relevance with Graves’ disease in 80%, and in patients with autoimmune hypothyroidism due to Hashimoto’s thyroiditis accounting for 10%, the remaining 10% of patients were individuals with no thyroid disease (Bartalena, Pinchera & Marcocci, 2000). However, the key proteins involved in TAO pathogenesis are still largely unclear.

Based on the fact that TAO is an autoimmune disorder, scholars have made great efforts to explore its primary antigen and how the immune system recognizes it (Heufelder, 2000). Up to now, it has been determined that autoimmunity is usually produced against the antigens common in the thyroid gland and the orbit, such as antigen TSH receptor (McGregor, 1998) and antigen collagen XIII (Wall & Lahooti, 2010). In addition to autoimmunity, several hypotheses have been put forth to explain the potential mechanism of ocular surface disease in TAO patients: environmental factors, such as stress, infectious agents, iodine, smoking, IFN and interleukin therapy, and sex steroids (Bahn, 2010; Sahli & Gunduz, 2017); genetic factors, such as thyroid-specific proteins and polymorphisms in protein genes affecting immune function, including CD40, CTLA 4, TG, IL-13, and IL-2RA (Chong et al., 2008; Wang & Smith, 2014). However, target proteins that can be effectively used in TAO immune targeted therapy are still scarce.

Normally, the ocular surface is maintained by tear, which mainly consists of mucins from the inner layer of the tear film (Royle et al., 2008), the aqueous portion (including dissolved mucins, water, and electrolytes), and the enveloped-effect lipid layer (Dartt, 2009). Both in TAO patients and healthy individuals, proteins are the most abundant components in tears, accounting for about 95% of its dry weight (Yang et al., 2020). Therefore, changes in tear protein composition can directly reflect the physiological and pathological state of the eye tissue. Fortunately, proteomic analysis and liquid chromatography-tandem mass spectrometry (LC-MS/MS) technology is a promising approach for cataloging the tear proteome and for searching potential biomarkers. However, the special skills of tear collection and technical limitations have hindered the progress of TAO tear proteome and its functional analysis. As a result, there are few reports on the TAO tear proteome, and a large gap in this field needs to be filled urgently.

In the present study, we aimed to investigate the difference in tear protein composition between the TAO patients and healthy controls (HC) using label-free comparative proteomics based on the Easy nano-scale LC-MS/MS, followed by a systematic data analysis to investigate the pathological mechanism about ocular surface damage in TAO.

## Materials and Methods

### Ethics statement

This study conformed to the ethical guidelines of the Declaration of Helsinki, and all protocols were approved by the Institutional Ethics Committee of the Changzheng Hospital affiliated to the Second Military Medical University of China (approval No.: 2015SLYS001). All subjects signed informed consent forms.

### Sample collection

#### Participants

A total of 30 TAO patients and 30 HC volunteers were included in this study. The general information of the enrolled subjects is shown in Table 1. The TAO patients were diagnosed and screened by two ophthalmologists with same opinion in Changzheng Hospital at active stage. Active stage was determined with the clinical active score ≥4 according to the previous study (European Group on Graves et al., 2006). Each patient and volunteer finished the clinical examination including ocular surface evaluation (such as eye protrusion, palpebral fissure height, number of blinks, tear film break up time, Schirmer I test), the Ocular Protection Index (OPI) (Ousler et al., 2008), the Ocular Surface Disease Index (OSDI) assessment and fluorescein staining.

**Table 1:** Details of samples used for this study.

	TAO group (*n* = 30)	HC group (*n* = 30)	*P* value
Age	22–57 (42.00 ± 11.91)	22–55 (40.27 ± 9.32)	0.376
Sex (M/F)	8/22	8/22	/
CAS	5 (4–8)	/	/
Ocular onset time (month)	8.5 (6, 18)	/	/
Thyroid dysfunction time (month)	15.5 (8, 25)	/	/
Thyroid dysfunction Hyper/Hypo/Nor	15/6/9	/	/

**Note:**

CAS, clinical active score; Ocular onset time, The time between the first symptom or sign of TAO and the diagnosis of TAO; Thyroid dysfunction time, The time between the discovery of thyroid dysfunction and diagnosis of TAO; Hyper/Hypo/Nor, Hyperthyroidism/ Hypothyroidism/Normal.

Thirty age- and sex-matched healthy persons who were euthyroid served as controls from the medical examination center in Changzheng Hospital. Patients and controls with additional disease or those who were taking medications that may affect the constitution of the tear film (eye drops, antispasmodics, diuretics, β-blockers, antihistamines, and so on) were not chosen for this study. All participants were required to stop using artificial tears 2 weeks before tear sampling.

#### Tear samples collecting

Tear samples were obtained from all participants during the same timeframe (3:00–5:00 pm) and at the same place (consulting room of department of ophthalmology in Changzheng hospital) by the same doctor. For each person, at least 10 μL of tear was collected from the lateral inferior conjunctival sac using calibrated glass micro capillary tubes without touching the ocular surface, minimizing irritation of the ocular surface or lid margin, without anesthesia. Tear samples were coded and snap-frozen at −80 °C.

### Tear samples preparation and label-free LC-MS/MS procedure

#### Tear samples preparation

For each group, the 30 tear samples (10 μL each) were blended and equally separated into three tubes. Each 100 μL tear sample was placed into a 10 kDa ultrafiltration tube and centrifuged at 14,000 g for 30 min at 4 °C. Then, trapped-precipitation in the filter was eluted by 100 μL 25 mM NH_4_HCO_3_ and centrifuged again at 14,000 g for 30 min at 4 °C. Subsequently, 80 μL SDT buffer was added into up layer to re-eluted trapped-precipitation, followed by vortexed and centrifuged at 14,000 g for 1 min at 4 °C. Finally, harvested protein from three repetitions was mixed and incubated in boiling water bath for 5 min for subsequent assays. Protein concentration of tear samples was quantified by the BCA kit (ab102536; Abcam, Cambridge, UK) according to the manufacturer’s instructions. Finally, about 20 μg proteins for each sample was analyzed by standard procedures of 10% SDS-PAGE to confirm parallelisms among samples.

#### Protein digestion

Digestion of protein (Each group had three repetitions) was performed following filter aided proteome preparation procedure described by Wisniewski et al. (2009). The detergent, dithiothreitol, was added to 200 μg of each sample with a final concentration of 100 mM, which was incubated in boiling water bath for 5 min, and then cool to room temperature. Then, the detergent and other low-molecular-weight components were removed using 200 μL uric acid (UA) buffer (8 M Urea, 150 mM trihydroxymethyl aminomethane-HCl Tris-HCl pH 8.0) by repeated ultrafiltration (Microcon units, 10 kD) facilitated by centrifugation at 14,000 g for 15 min, following added 100 μL iodoacetamide (50 mM in UA) for blocking reduced cysteine residues. Next, all samples were vortexed at 600 rpm for 1 min and incubated at 25 °C in dark for 30 min and removed supernatant by centrifuging at 14,000 g for 10 min. The filter was washed with 100 μL UA buffer and repeated twice, and then washed with 100 μL 25 mM ammonium bicarbonate solution and repeated twice. Then, 40 μL Trypsin buffer (4 μg Trypsin in 25 mM NH_4_HCO_3_) was added and vortexed at 600 rpm for 1 min, and then incubated at 37 °C for 16–18 h. Finally, the resulting peptides were collected as a filtrate and the peptide concentrations from the filtrate were determined by OD280.

#### LC - electrospray ionization (ESI) MS/MS analysis by Q Exactive

The peptide of three repeated samples were desalted on C18 Cartridges (Empore™ SPE Cartridges C18 (standard density), bed I.D. 7 mm, volume 3 ml; Sigma, St. Louis, MI, USA), then concentrated by vacuum centrifugation and reconstituted in 40 µL of 0.1% (v/v) trifluoroacetic acid. Firstly, Easy nLC1000 was used for separation. Liquid phase A is 0.1% formic acid aqueous solution, liquid phase B is 0.1% formic acid acetonitrile aqueous solution (acetonitrile is 84%). The C18 column (Thermo EASY column SC001, 150 μm × 100 mm) was balanced with 95% liquid phase A. Samples were loaded to C18 column (Thermo EASY column SC001, 20 mm inner diameter, 5 μm resin, 100 μm long) by automatic sampler and separated by a C18 column (Thermo EASY column, 75 μm inner diameter, 3 μm resin, 10 cm long) at a flow rate of 250 nL/min controlled by IntelliFlow technology over 60 min. The liquid phase gradient was as follows: 0–50 min, linear gradient of liquid B from 0 % to 50%; 50–54 min, linear gradient of liquid phase B from 50% to 100%; 54–60 min, liquid phase B maintained at 100%.

Next, after separation by capillary HPLC, samples were analyzed by ESI MS on Q Exactive (Thermo Finnigan, San Jose, CA, USA). The MS method detected positive ion and primaquine (parent ion) from the survey scan (300–1,800 m/z). MS1 survey scans were acquired at a resolution of 70,000 at m/z 200 and predictive AGC target was 3e6, maximum IT was 10 ms; the number of scan ranges was 1 and dynamic exclusion was 20.0 s. The mass–charge ratios of polypeptide and polypeptide fragments were collected according to the following methods: Each full scan was followed by 10 debris maps (MS2 scan) for higher-energy collision dissociation with 2 m/z isolation window; a resolution of 17,500 at m/z 200; microscans was 1; maximum IT was 60 ms; normalized collision energy was 27 eV; Underfill ratio was 0.1%.

An automatic database search against the Uniport, saved as uniport_human.fasta (133,549 total entries, downloaded 03/08/2013), using mascot software was applied on LC-ESI data for protein identification. ProteomicsTools was 3.1.6. The searching parameters were set as follows: trypsin was specified as the proteolytic enzyme, two missed cleavage site per peptide was allowed, carbamidomethyl C was set as the fixed modification and oxidation M was set as the variable modification. Peptides tolerance was 20 ppm while ms/ms tolerance was 0.1 Da. The cutoff of global false discovery rate for peptide and protein identification was set to 0.01.

#### LC-MS/MS analysis by Q exactive

The peptide of three repeated samples was desalted on C18 Cartridges (Empore™ SPE Cartridges C18 (standard density), bed I.D. 7 mm, volume 3 ml, Sigma, St. Louis, MI, USA), then concentrated by vacuum centrifugation and reconstituted in 40 µL of 0.1% (v/v) trifluoroacetic acid. The 5 μg of each digested protein sample (repeated replicates) was loaded onto LC-MS/MS. Firstly, Easy nLC1000 was used for separation. Liquid phase A is 0.1% formic acid acetonitrile aqueous solution (acetonitrile is 2%), and liquid phase B is 0.1% formic acid acetonitrile aqueous solution (acetonitrile is 84%). The C18-reversed-phase trap column (Thermo EASY column SC001, 150 μm × 100 mm) was balanced with 100% liquid phase A. Samples were loaded to C18-reversed-phase trap column (Thermo EASY column SC001, 150 μm × 20 mm) by an automatic sampler and separated by column at a flow rate of 400 nL/min controlled by IntelliFlow technology over 120 min. The liquid phase gradient was as follows: 0–100 min, linear gradient of liquid B from 0% to 45%; 100–108 min, linear gradient of liquid phase B from 45% to 100%; 108–120 min, liquid phase B maintained at 100%.

Next, after separation by capillary HPLC, samples were analyzed by Q-Exactive MS. MS data was acquired using a data-dependent top-10 method dynamically choosing the most abundant precursor ions from the survey scan (300–1,800 m/z) for higher energy collision dissociation fragmentation. MS1 survey scans were acquired at a resolution of 70,000 at m/z 200 and MS2 resolution for higher energy collision dissociation spectra was set to 17,500 at m/z 200. MS experiments were performed triply for each sample.

MaxQuant software version 1.3.0.5 was used in the MS data analysis. MS data were searched against the Uniport, saved as uniport_human.fasta (133,549 total entries, downloaded 03/08/2013). A main search was set at a precursor mass window of 6 ppm. The search followed an enzymatic cleavage rule of Trypsin/P and allowed maximal two missed cleavage sites and a mass tolerance of 20 ppm for fragment ions. The cutoff of global false discovery rate for peptide and protein identification was set to 0.01. Label-free quantification was carried out according to MaxQuant (Matheis et al., 2012; Okrojek et al., 2009). Intensity-based absolute quantification in MaxQuant was performed on the identified peptides to quantify protein abundance.

### Differential protein analysis

The data searched from MaxQuant were analyzed with Perseus software version 1.3.0.4. The differential expression analysis of proteins between TAO group and HC group was determined by student’s t-test, on the SPSS software version 13.0. Each pair of tubes (one from the TAO group and the other from the HC group) was handled with t-test, the results of the three t-tests were averaged. Only when a certain protein was expressed in at least two samples of the TAO group and at least two samples of the HC group at the same time, it was allowed to perform a different abundance protein (DAP) analysis. When protein abundance between two groups meets the fold-change (FC; TAO/HC) >4 and *P* < 0.05, it was considered a significant DAP. Finally, the analysis of Gene Ontology (GO, http://geneontology.org/), and KEGG (https://www.genome.jp/kegg/pathway.html) were applied on DEPs.

### Statistical analysis

For data from clinical examination, data were tested for normal distribution using Shapiro-Wilk (W test). Normally distributed data were expressed as mean ± standard deviation, and non-normally distributed data were expressed as median (interquartile range). After each observation data was tested for normality, Dennett’s T test was used for intragroup comparison of data that conformed to the normal distribution; data that were not normally distributed were compared using the Wilcoxon signed rank sum test for paired-sample comparisons.

## Results

### Comparison of ocular measurement indices

We first characterized the changes of ocular surface damage in TAO patients, and the results were shown in Table 2. Compared with the HC group, the indices of eye protrusion, palpebral fissure height, number of blinks, and Ocular Surface Disease Index significantly increased in the TAO group, while indices tear film break up time, the Schirmer I test, and the Ocular Protective Index all significantly decreased. These results indicate that the ocular surface was severely damaged in the TAO group.

**Table 2:** Compares the seven ocular measurement indices. Normally distributed data were expressed as mean ± standard deviation, and non-normally distributed data were expressed as median (interquartile range). Dennett’s T test was used for intragroup comparison of data that conformed to the normal distribution; data that were not normally distributed were compared using the Wilcoxon signed rank sum test for paired-sample comparisons.

Index	HC	TAO	*P*-value
Eye protrusion (mm)	13.34 ± 1.71	20 (18, 21)	z = −4.677, 0.000
Palpebral fissure height (mm)	8.00 ± 0.78	11.06 ± 2.59	t = 6.905, 0.000
Number of blinks	16 (14, 18)	30 (25, 44)	z = 7.652, 0.000
Tear film break up time (s)	9 (8, 11)	2 (2, 3.75)	z = −8.038, 0.000
Schirmer I test (mm)	14.27 ± 2.81	9.51 ± 4.23	t = −5.993, 0.000
Ocular Surface Disease Index	21.67 ± 8.53	38.43 ± 20.54	t = 4.925, 0.000
Ocular Protective Index	2.53 (2.00, 2.85)	1.25 (0.87, 1.60)	z = −5.648, 0.000

### Quality control of tear proteins

**Figure 1:**
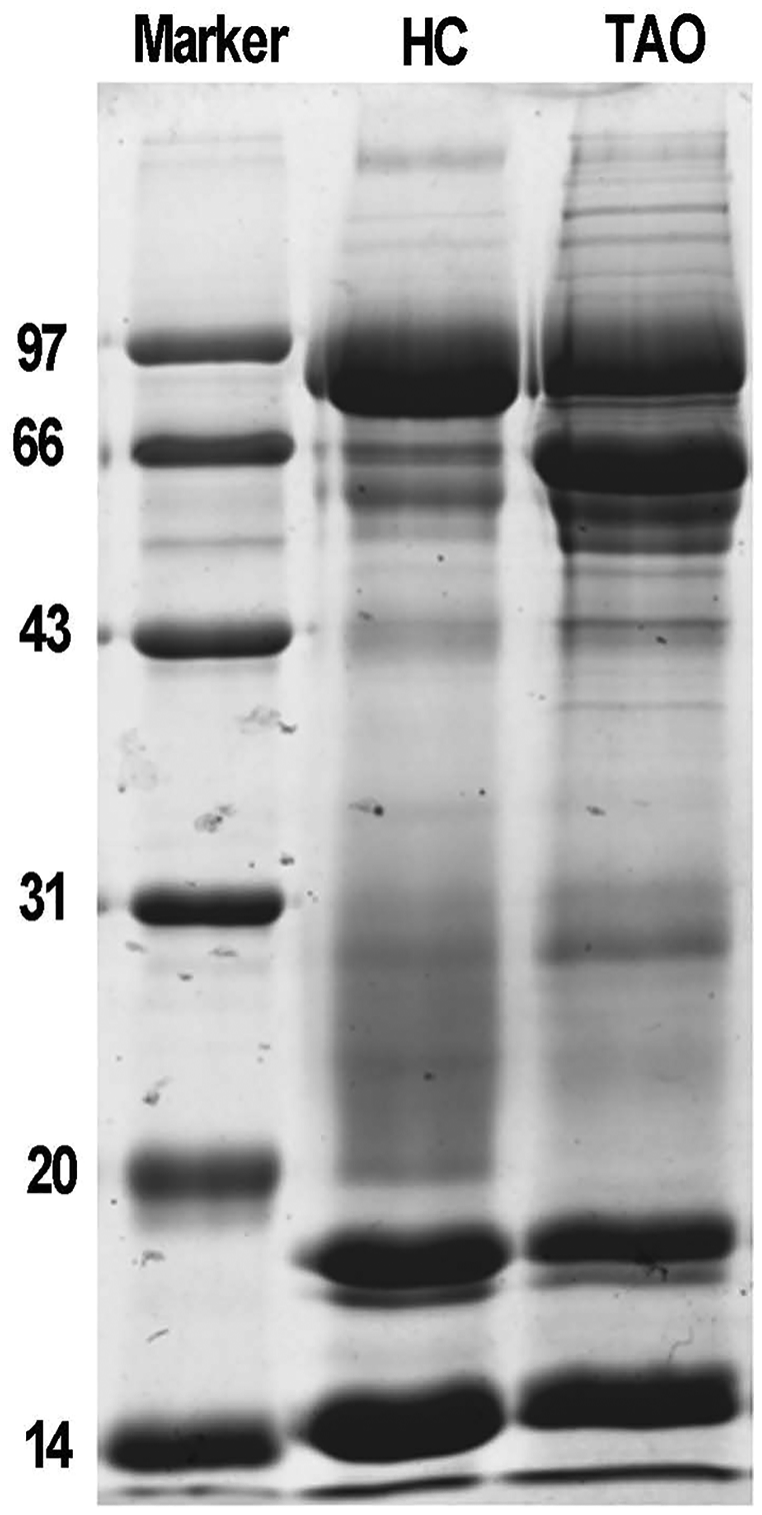
Sample quality control and parallel testing. Gel image of SDS-PAGE.

Next, total protein of tears was extracted for proteomic analysis. Isolated protein concentration of tear samples was determined by BCA kit, as shown in Table S1, the protein concentration of the TAO group and the HC group was 11.3 and 14.6 μg/μL, respectively. The data suggested that isolated proteins were of sufficient quality for subsequent experiments. The difference in protein composition between the two groups was characterized by SDS-PAGE for the first time. It is evident from Fig. 1, compared with the HC group, a dramatic increase of the protein band around 66 kDa was observed in the TAO group, as well as slight increase between 20–30 kDa. These results implicated that parallelism among samples was good and tear protein composition in TAO patients was changed.

### LC-ESI MS/MS tear proteome analysis

**Figure 2:**
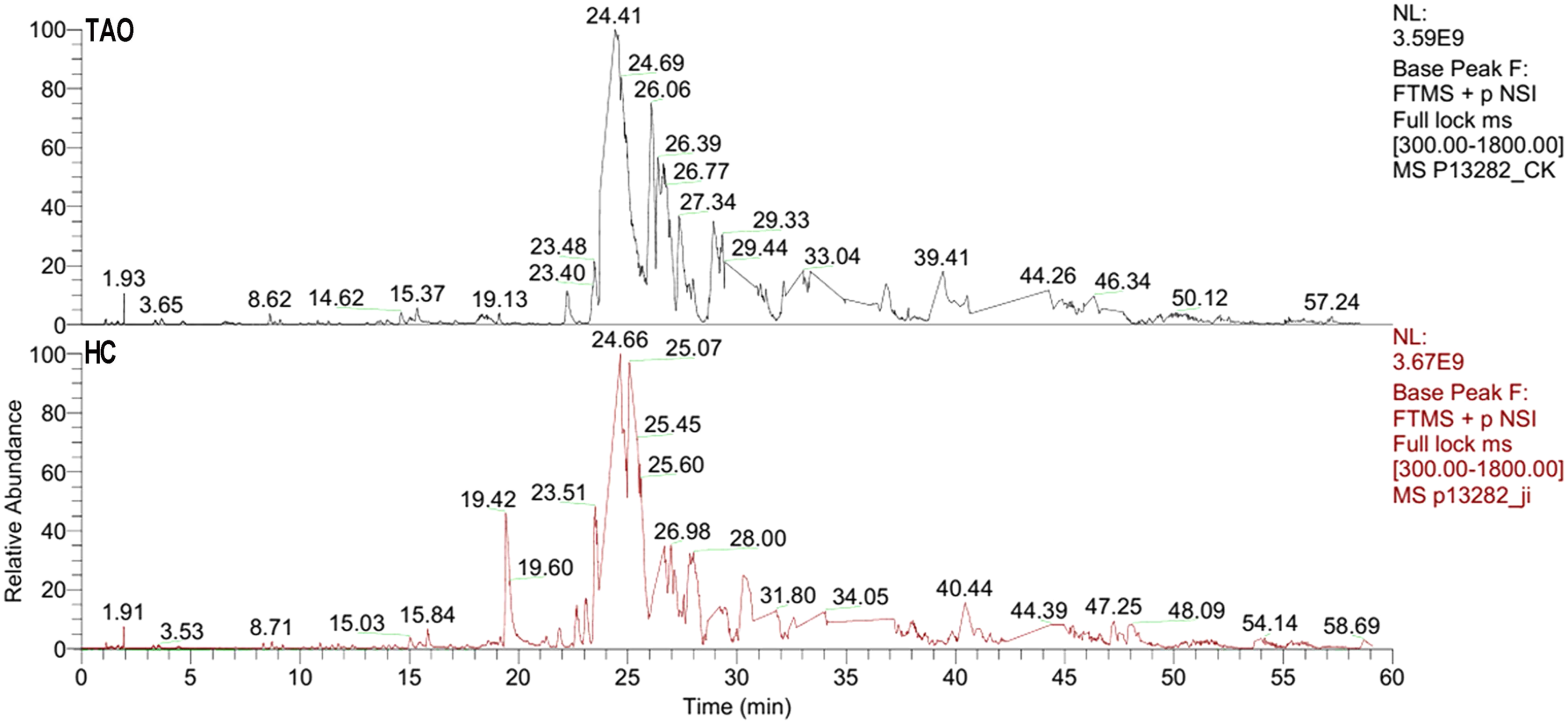
Base peak of ESI mass spectra of tear proteins of the TAO group and the HC group.

The six tear samples were first pre-examined by LC-ESI MS/MS. As shown in Fig. 2, a 60 min gradient was applied and high quality separation and detection were achieved. A total of 125 and 181 protein group was identified in the HC group and the TAO group, respectively. After filtering by unique peptide ≥2, remaining 59 and 104 protein group was identified in the HC group and the TAO group, respectively. In addition, the ESI mass spectra test also confirmed parallelisms among samples. Thus, the results of parallelism and identification efficiency of LC-ESI MS/MS were good enough for encouraging us further later experiments.

### Protein profiling of LC-MS/MS

**Figure 3:**
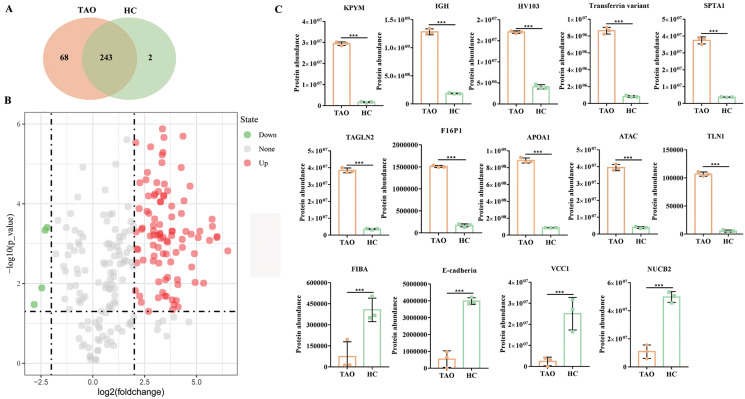
Overall of protein profiles. (A) Venn diagram of the number of identified proteins in TAO group and HC group. (B) Volcanic map of DAPs between TAO group and HC group. (C) Histogram of protein abundance of top 10 up-regulated DAPs and four down-regulated DAPs detected by LC-MS/MS.

Subsequently, six tear samples were digested and subjected to Easy nLC-MS/MS analysis. In this study, a total of 313 proteins were identified from the two groups of pooled tear samples (Table S2), and 243 proteins were shared in the TAO and the HC group. Besides, 68 and two unique proteins (namely myosin-reactive immunoglobulin heavy chain variable region and anti-streptococcal/anti-myosin immunoglobulin lambda light chain variable region) were identified in the TAO and the HC groups, respectively (Fig. 3A, Table S2). Only 226 proteins were simultaneously detected in at least two samples from the TAO group and the HC group. In this study, only these 226 proteins were allowed into the DAP analysis.

Using a 4-FC in a protein abundance as a benchmark for significant change, and thus a total of 103 DAPs were reliably quantified by intensity-based absolute quantification analysis. Among these 103 DAPs, 99 DAPs were up-regulated in TAOs group, compared with HC group (Fig. 3B, Table S3). The top 10 significantly up-regulated DAPs and all four down-regulated DAPs in tears between TAO *vs* HC were shown in Fig. 3C and Table 2. Interestingly, these significant changes in DAP were mainly related to lipocalin and immunity, such as fructose-1, 6-bisphosphatase 1 (F16P1), apolipoprotein A-I (APOA1), Actin, alpha cardiac muscle 1 (ATAC), transferrin variant, and Ig heavy chain V-I region V35 (HV103). Moreover, compared with HC group, 4 DAPs were down-regulated in TAOs group, including nucleobindin-2 (NUCB2), E-cadherin, fibrinogen alpha chain (FIBA), and VEGF co-regulated chemokine 1 (VCC1) (Fig. 3C and Table 3).

**Table 3:** The top 10 significantly up-regulated and all four down-regulated DAPs of tears between TAO *vs* HC.

ACC no.	Protein name	Log_2_FC	*P* value
Up-regulated			
P09467	Fructose-1,6-bisphosphatase 1 (F16P1)	3.1513	5.67E−07
P14618	Pyruvate kinase isozymes (KPYM)	4.1576	6.53E−07
P02647	Apolipoprotein A-I (APOA1)	3.3560	1.32E−06
Q9Y490	Talin-1 (TLN1)	4.3478	2.01E−06
P37802	Transgelin-2 (TAGLN2)	3.4277	2.16E−06
P23083	Ig heavy chain V-I region V35 (HV103)	2.0699	2.92E−06
Q6GMX6	IGH@ protein (IGH)	2.7893	3.71E−06
Q53H26	Transferrin variant	3.3814	5.13E−06
P68032	Actin, alpha cardiac muscle 1 (ATAC)	3.3449	5.88E−06
P02549	Spectrin alpha chain, erythrocytic 1 (SPTA1)	3.3311	9.28E−06
Down-regulated			
P80303	Nucleobindin-2 (NUCB2)	−2.2037	3.93E−04
Q9UII7	E-cadherin	−2.3007	4.60E−04
P02671	Fibrinogen alpha chain (FIBA)	−2.4627	1.29E−02
Q6UXB2	VEGF co-regulated chemokine 1 (VCC1)	−2.8258	3.36E−02

### GO enrichment analysis of DAPs

**Figure 4:**
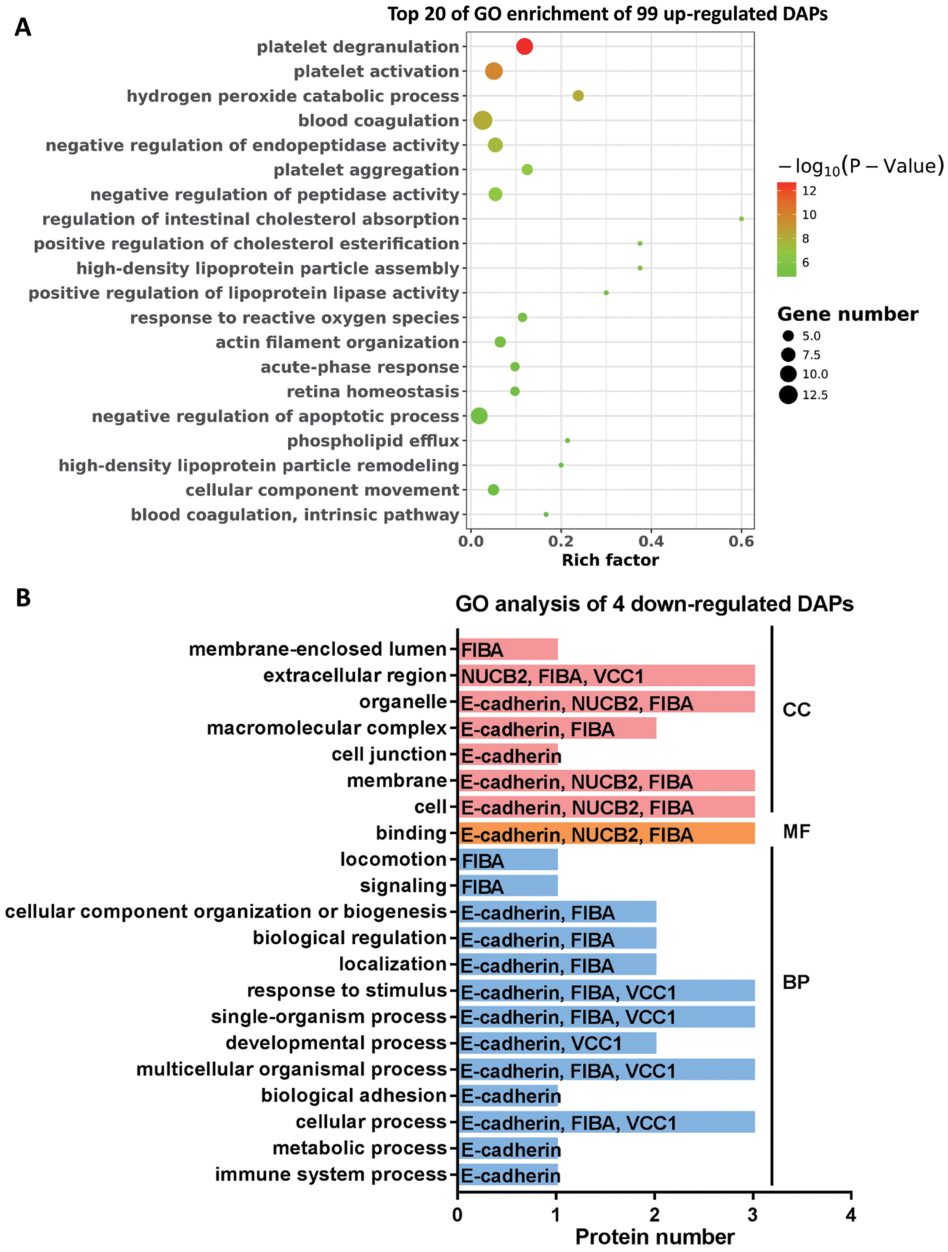
Go analysis of DAPs. (A) Top 20 of Go enrichment of 99 up-regulated DAPs. Red to green indicates that the GO entry has increased in significance. The size of the circle indicates the number of proteins. (B) GO analysis of four down-regulated DAPs. Red represents cellular components (CC), yellow represents molecular functions (MF), and blue represents biological processes (BP).

To further explore the molecular functions involved in the alteration of tear protein components induced by TAO disease, we performed GO analysis on up-regulated and down-regulated DAPs. According to the results of GO enrichment, as shown in Fig. 4A, TAO-induced up-regulation of DAPs were mainly involved in retina homeostasis and GO entries related to platelet function (such as platelet degranulation, platelet activation, platelet aggregation, blood coagulation, and blood coagulation intrinsic pathway) and lipid metabolism (such as regulation of intestinal cholesterol absorption, positive regulation of cholesterol esterification, high-density lipoprotein particle assembly, phospholipid efflux, and high-density lipoprotein particle remodeling). Therefore, we hypothesized that the changes in the lipid composition of tears may cause the loss of water-locking ability and damage stability of tears, and thus lead to TAO disease; in addition, excessive encapsulating of platelet-related proteins into tear fluid also implicates the metabolism dysfunction of ocular in TAO patients.

Moreover, it can be seen from Fig. 4B that the four DAPs significantly down-regulated in TAO group have highly similar molecular functions, they were mainly involved in cellular-related processes and junction, such as cellular process, multicellular organismal process, single-organism process, binding, membrane, cell junction, and extracellular region. These results indicated that cellular metabolic process was disturbed in TAO patients.

### KEGG analysis of DAPs

**Figure 5:**
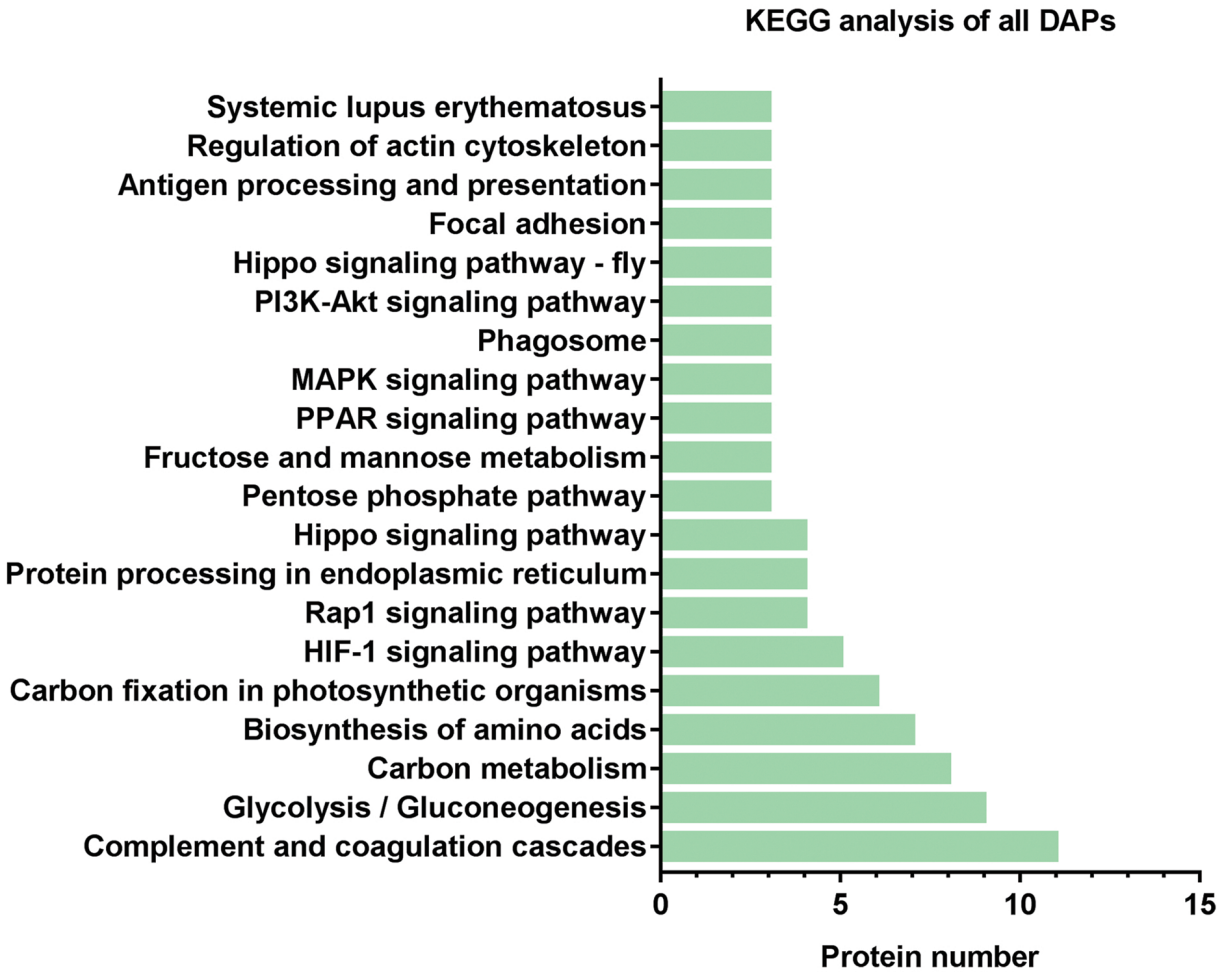
KEGG analysis of all DAPs. The vertical axis represents the name of the pathway, and the horizontal axis represents the number of proteins clustered in pathway.

**Figure 6:**
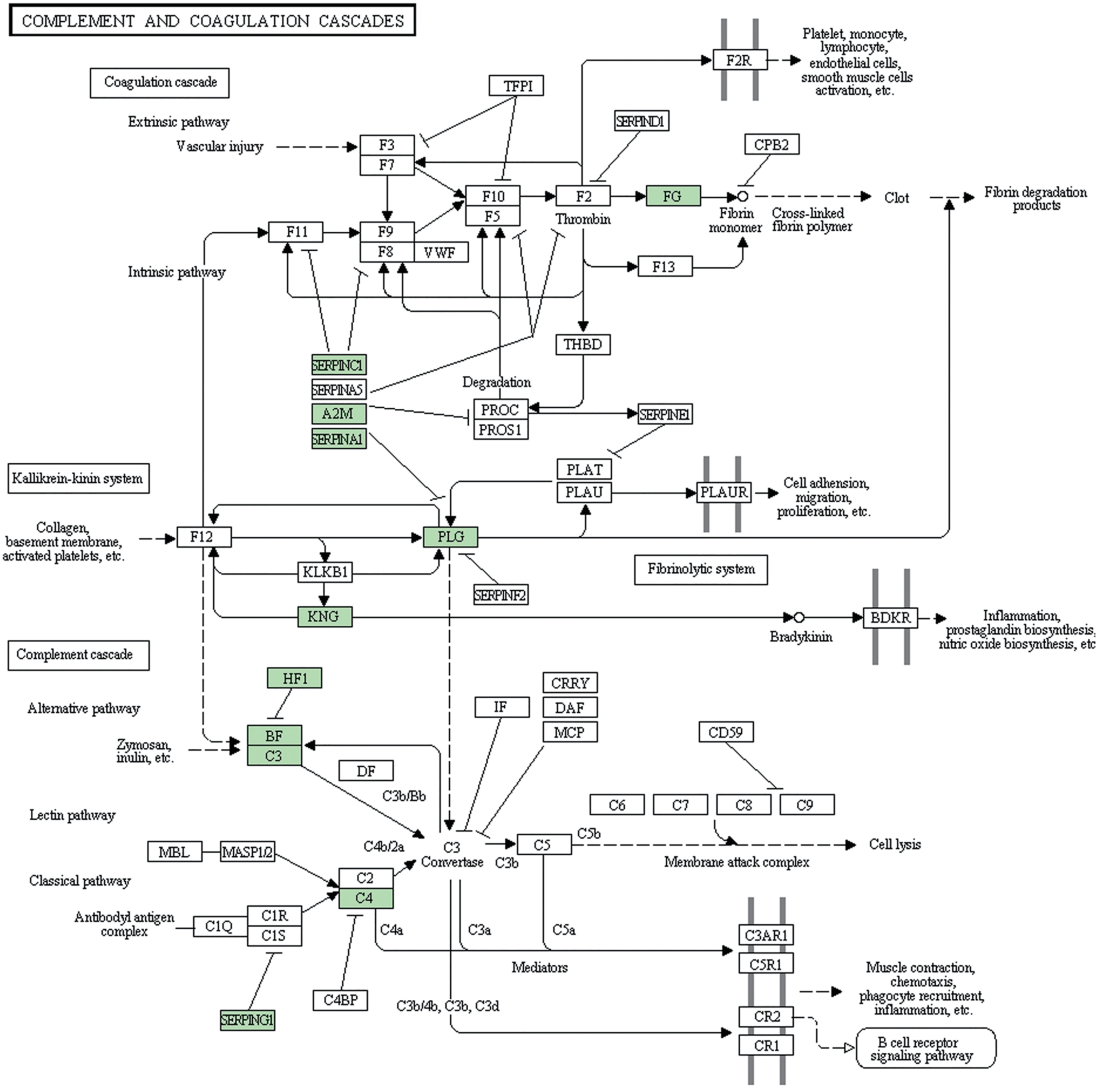
Complement and coagulation cascades pathway. DAPs were shown up in bright green.

Subsequently, we further analyzed the molecular pathways involved in DAPs. KEGG results indicated that DAPs were enriched in 117 signal transduction pathways, and we listed the top 20 pathways (Fig. 5). KEGG pathway indicated that the DAPs were mainly involved in immune-related pathways, including systemic lupus erythematosus, antigen processing and presentation, phagosome, the PPAR signaling pathway, MAPK signaling pathway, PI3K-Akt signaling pathway, Rap1 signaling pathway, and complement and coagulation cascades (Fig. 4). Among these pathways, the most enriched pathway was the complement and the coagulation cascades pathway. A total of 11 DAPs were involved in complement and the coagulation cascades, such as Complement C3 (C3), C4, and alpha-1-antitrypsin (SERPINA1) and antithrombin-III (SERPINC1) (Fig. 6). Therefore, we speculated that the immune-related pathway alteration might be one of the functional reasons that mediates TAO progression.

## Discussion

Tears are one of the most critical body fluids in the human body, it main functions include lubrication for the cornea, refraction, providing nutrition for ocular cells, and resistance to the invasion of various pathogens. Although the amount of tear fluid is very small, tear fluid contains extremely complex biological components including proteins, electrolytes, lipids, and small molecule metabolites (von Thun Und Hohenstein-Blaul, Funke & Grus, 2013). The study reported that protein is the most abundant component in tears, accounting for about 95% of its dry weight (Yang et al., 2020). Therefore, the use of tear proteomics to clarify the pathogenesis of eye-related diseases has become a research hotspot. Here, visible defects at the ocular surface were observed in TAO patients, and this study is the first record of label-free quantitative nLC-MS/MS employed to identify DAPs in tear fluids between TAO patients and HC subjects.

In the present study, the top 10 significantly up-regulated DAPs were mainly related to lipocalin and immunity, such as transferrin variant, HV103, and APOA1. The transferrin family member, lactoferrin, is one of the four major components of tear fluid, along with lysozyme, lipocalin, and secretory immunoglobulin A (sIgA) (Molloy et al., 1997). Lactoferrin can regulate the activity of macrophages and stimulate the synthesis of lymphocytes, and can also bind to receptors on the surface of immune cells, thereby exerting immune regulation functions (Pierce & Legrand, 2009). Consistent with our results, Baker et al. also found that the lactoferrin abundance was increased in patients with Graves’s ophthalmopathy (Baker et al., 2006). HV103 is an immunoglobulin, and immunoglobulin up-regulation indicates the involvement of immune factors and the occurrence of inflammation (Markina et al., 2020). Besides, APOA1 is the main protein of the high-density lipoproteins and is involved in inflammatory processes (Fernandez-Sendin et al., 2020). Therefore, we speculate that the significantly up-regulated DAPs may lead to TAO progression through mediating inflammation and abnormal lipid metabolism. Clinical improvement of TAO may be achieved by targeting the abnormal expression of these DAPs.

Only four down-regulated DAPs in TAO tears were identified compared with HC group, namely NUCB2, E-cadherin, FIBA, and VCC1. The physiological function of NUCB2 (Tao et al., 2020), E-cadherin (Kaszak et al., 2020), FIBA (Chen et al., 2019), and VCC1 (Li et al., 2005) were related to mediating cell adhesion and maintaining the integrity of cell/tissue structure, or immunity. Therefore, the down-regulation of the 4 DAPs in this study suggests the loss of tear film adhesion and stability in the TAO patients.

Lytic immunity of the complement system is known as one of the anti-infection mechanisms of human body. Normal tissues associated with target cells may also get damaged in this process. So the activation of the complement system could either help to resist pathogens or to damage autologous tissue. Many studies have proved that complement inhibitors can be used in the treatment of various ocular diseases. For example, Charbel Issa et al. (2005) reviewed the evidence of complement system possibly applied in age-related macular degeneration through immunepathogenic processes. Sohn et al. (2000) evidenced that the complement system is continuously active at a low level in the normal eye and systemic complement depletion by cobra venom factor inhibited the progression of anterior uveitis. Annamalai et al. (2021) described that target complement system inhibitor reduced the ocular injury in retinal degeneration model. In our study, complement and coagulation cascades pathway is the most DAPs enriched pathway, containing C3 and C4, and SERPINA1. It is reported that 40% of TAO patients have complement cascades activation, such as C3 deposition (Antonelli et al., 1996). Copy number variation of C4 (Liu et al., 2011) and DNA methylation of SERPINA1 (Cai et al., 2021) were associated with Graves’ disease. These results implicate that the complement system component might be overactive in TAO patients. Therefore, developing a novel inhibitor that targets these DAPs involved in complement and coagulation cascades may be a promising treatment strategy in TAO.

Furthermore, we found that DAPs participated in several “star pathways” related to disease and immunity, such as the PI3K-Akt signaling pathway. A study reported that during Spica Prunellae employed in TAO therapy, PI3K-AKT signaling pathway plays a central role in anti-TAO system (Zhang et al., 2020), suggesting the potential value of the PI3K-AKT signaling pathway in TAO treatment. Moreover, Graves’ disease is considered as the consequence of a breakdown in TSHR tolerance, and selected cleavage TSHR antibody could produce a robust effect on the MAPK/PI3K-AKT/mTOR/S6K signaling cascades, the maintenance of which determined the thyroid cell fate of death (Morshed & Davies, 2015). Importantly, an inhibitor of the PI3K-Akt signaling pathway, wortmannin, could attenuate thyroid injury associated with severe acute pancreatitis in rats (Abliz et al., 2015). Taken together, these results prompted us to speculate that targeted inhibition of the PI3K-Akt signaling pathway may be a potential therapeutic strategy for TAO as well as other thyroid-related diseases.

## Conclusions

In conclusion, this study is the first record of label-free quantitative nLC-MS/MS employed to identify DAPs in tear fluids between TAO patients and HC. A total of 103 DAPs were identified including 99 up-regulated DAPs and four down-regulated DAPs in TAO group compared with HC group. TAO induced up-regulated expression of proteins related to lipid metabolism and platelet molecular function and down-regulated expression of proteins involved in cellular processes. The DAPs were mainly involved in immune-related pathways, especially concentrated in complement and coagulation cascades pathway. This study provides several proteins and pathways altered in the HC from TAO and provides potential molecular targets for further study on the precise mechanism of pathogenesis of TAO.

## Supplemental Information

10.7717/peerj.13701/supp-1 Supplemental Information 1 The protein content of samples and peptides OD280 value.

10.7717/peerj.13701/supp-2 Supplemental Information 2 Detailed information on 313 proteins identified.

10.7717/peerj.13701/supp-3 Supplemental Information 3 List of 103 DAPs of tears between TAO group and HC group.

10.7717/peerj.13701/supp-4 Supplemental Information 4 Raw data of information of subjects.

## Additional Information and Declarations

### Data Availability

The following information was supplied regarding data availability: Raw data is available in the Supplemental Information.
