# Supplementary material for: Characterization of human tear proteome reveals differentially abundance proteins in thyroid-associated ophthalmopathy
Source: PeerJ. 2022 Jul 12;10:e13701. doi: 10.7717/peerj.13701 (PMC9285480; doi:10.7717/peerj.13701)
Supplement: Supplemental Information 1 [file peerj-10-13701-s001.docx]

## **Supplemental Table 1. The protein content of samples and peptides OD280 value**

|  | Protein concentration  (μg/μL) / Total (μL) | Peptides concentration (μg/μL) |
| --- | --- | --- |
| Normal | 14.6/75 | 2.41 |
| TAO | 11.3/75 | 2.02 |
