# Supplementary material for: Characterization of human tear proteome reveals differentially abundance proteins in thyroid-associated ophthalmopathy
Source: PeerJ. 2022 Jul 12;10:e13701. doi: 10.7717/peerj.13701 (PMC9285480; doi:10.7717/peerj.13701)
Supplement: Supplemental Information 3 [file peerj-10-13701-s003.docx]

**Supplemental Table 3. List of 103 DAPs of tears between TAO group and HC group.**

| **ID** | **Protein name** | **Mol. weight [kDa]** | **Fold change**  **TAO/HC** | **t-test**  **P value** |
| --- | --- | --- | --- | --- |
| P09467 | Fructose-1,6-bisphosphatase 1 | 36.842 | 8.8844 | 0.0000 |
| P14618 | Pyruvate kinase isozymes M1/M2 | 57.936 | 17.8469 | 0.0000 |
| P02647 | Apolipoprotein A-I | 30.777 | 10.2387 | 0.0000 |
| Q9Y490 | Talin-1 | 269.76 | 20.3616 | 0.0000 |
| P37802 | Transgelin-2 | 22.391 | 10.7606 | 0.0000 |
| P23083 | Ig heavy chain V-I region V35 | 13.009 | 4.1987 | 0.0000 |
| Q6GMX6 | IGH@ protein | 51.082 | 6.9130 | 0.0000 |
| Q53H26 | Transferrin variant (Fragment) | 77.079 | 10.4211 | 0.0000 |
| P68032 | Actin, alpha cardiac muscle 1 | 42.019 | 10.1604 | 0.0000 |
| P02549 | Spectrin alpha chain, erythrocytic 1 | 280.01 | 10.0640 | 0.0000 |
| G9K388 | YWHAE/FAM22A fusion protein (Fragment) | 41.224 | 19.1689 | 0.0000 |
| P17066 | Heat shock 70 kDa protein 6 | 71.027 | 6.2064 | 0.0000 |
| P63261 | Actin, cytoplasmic 2 | 41.792 | 5.8963 | 0.0000 |
| P06733 | Alpha-enolase | 47.168 | 8.4908 | 0.0000 |
| P10599 | Thioredoxin | 11.737 | 4.8187 | 0.0000 |
| P01009 | Alpha-1-antitrypsin | 46.736 | 10.9331 | 0.0000 |
| P01861 | Ig gamma-4 chain C region | 35.94 | 25.7092 | 0.0000 |
| P02790 | Hemopexin | 51.676 | 12.1676 | 0.0000 |
| P08294 | Extracellular superoxide dismutase [Cu-Zn] | 25.851 | 8.9749 | 0.0000 |
| P04406 | Glyceraldehyde-3-phosphate dehydrogenase | 36.053 | 7.9413 | 0.0001 |
| A6XGL1 | Transthyretin | 20.199 | 10.6659 | 0.0001 |
| P26447 | Protein S100-A4 | 11.728 | 7.8188 | 0.0001 |
| P01024 | Complement C3 | 187.15 | 6.4084 | 0.0001 |
| Q06830 | Peroxiredoxin-1 | 22.11 | 10.7113 | 0.0001 |
| P04217 | Alpha-1B-glycoprotein | 54.253 | 21.0454 | 0.0001 |
| P09211 | Glutathione S-transferase P | 23.356 | 9.6906 | 0.0001 |
| P21291 | Cysteine and glycine-rich protein 1 | 20.567 | 9.5106 | 0.0001 |
| Q5W0H4 | Translationally-controlled tumor protein | 21.525 | 13.9416 | 0.0001 |
| P05109 | Protein S100-A8 | 10.834 | 4.6966 | 0.0001 |
| P55072 | Transitional endoplasmic reticulum ATPase | 89.321 | 6.1154 | 0.0002 |
| P14550 | Alcohol dehydrogenase [NADP(+)] | 36.573 | 6.4963 | 0.0002 |
| P20962 | Parathymosin | 11.53 | 19.2915 | 0.0002 |
| P60174 | Triosephosphate isomerase | 30.791 | 13.9722 | 0.0002 |
| B4E1B2 | cDNA FLJ53691, highly similar to Serotransferrin | 74.831 | 7.8926 | 0.0002 |
| D3DRR6 | Inter-alpha (Globulin) inhibitor H2, isoform CRA_a | 106.57 | 53.7752 | 0.0002 |
| P19652 | Alpha-1-acid glycoprotein 2 | 23.602 | 9.3327 | 0.0003 |
| P00352 | Retinal dehydrogenase 1 | 54.861 | 7.5674 | 0.0004 |
| P01042 | Kininogen-1 | 71.957 | 54.4566 | 0.0004 |
| B3KS79 | cDNA FLJ35730 fis, clone TESTI2003131, highly similar to alpha-1-antichymotrypsin | 50.628 | 5.2282 | 0.0004 |
| P30101 | Protein disulfide-isomerase A3 | 56.782 | 5.1162 | 0.0004 |
| B4DR52 | Histone H2B | 18.041 | 7.6124 | 0.0005 |
| P30044 | Peroxiredoxin-5, mitochondrial | 22.086 | 14.2641 | 0.0005 |
| P22626 | Heterogeneous nuclear ribonucleoproteins A2/B1 | 37.429 | 35.7731 | 0.0005 |
| P02652 | Apolipoprotein A-II | 11.175 | 14.0478 | 0.0005 |
| H7C2N1 | Thymosin alpha-1 (Fragment) | 15.747 | 22.4385 | 0.0005 |
| P01625 | Ig kappa chain V-IV region Len | 12.64 | 4.2554 | 0.0005 |
| P02787 | Serotransferrin | 77.063 | 9.4298 | 0.0005 |
| P01008 | Antithrombin-III | 52.602 | 26.7555 | 0.0006 |
| P68104 | Elongation factor 1-alpha 1 | 50.14 | 9.3718 | 0.0006 |
| P25815 | Protein S100-P | 10.4 | 7.6527 | 0.0006 |
| Q15651 | High mobility group nucleosome-binding domain-containing protein 3 | 10.666 | 8.5891 | 0.0006 |
| O75368 | SH3 domain-binding glutamic acid-rich-like protein | 12.774 | 13.9386 | 0.0006 |
| P08603 | Complement factor H | 139.09 | 6.8188 | 0.0006 |
| P68871 | Hemoglobin subunit beta | 15.998 | 63.4931 | 0.0007 |
| P01023 | Alpha-2-macroglobulin | 163.29 | 10.4758 | 0.0007 |
| Q59EJ3 | Heat shock 70kDa protein 1A variant (Fragment) | 77.495 | 63.0385 | 0.0007 |
| P30740 | Leukocyte elastase inhibitor | 42.741 | 33.8174 | 0.0007 |
| Q9H4A4 | Aminopeptidase B | 72.595 | 9.6407 | 0.0007 |
| E9PGN7 | Plasma protease C1 inhibitor | 59.491 | 19.8263 | 0.0008 |
| P69905 | Hemoglobin subunit alpha | 15.257 | 49.3208 | 0.0008 |
| P02763 | Alpha-1-acid glycoprotein 1 | 23.511 | 11.8304 | 0.0008 |
| D6RF35 | Vitamin D-binding protein | 53.02 | 12.1389 | 0.0009 |
| P06703 | Protein S100-A6 | 10.18 | 7.5190 | 0.0011 |
| P21980 | Protein-glutamine gamma-glutamyltransferase 2 | 77.328 | 8.8848 | 0.0012 |
| Q4LE82 | C4A variant protein (Fragment) | 193.44 | 19.9461 | 0.0012 |
| Q5NV82 | V4-2 protein (Fragment) | 11.142 | 4.3462 | 0.0013 |
| P30041 | Peroxiredoxin-6 | 25.035 | 4.5067 | 0.0014 |
| Q01469 | Fatty acid-binding protein, epidermal | 15.164 | 90.8801 | 0.0015 |
| B4E1Z4 | Complement factor B | 140.94 | 16.1109 | 0.0017 |
| Q6P5V6 | SNX5 protein (Fragment) | 47.438 | 10.5008 | 0.0018 |
| P02749 | Beta-2-glycoprotein 1 | 38.298 | 29.7547 | 0.0018 |
| B7Z8Q2 | cDNA FLJ55606, highly similar to Alpha-2-HS-glycoprotein | 46.626 | 11.6582 | 0.0018 |
| Q59G88 | Coronin (Fragment) | 59.613 | 7.7709 | 0.0020 |
| P06702 | Protein S100-A9 | 13.242 | 5.3752 | 0.0026 |
| B4DE31 | cDNA FLJ54957, highly similar to Transketolase (EC 2.2.1.1) | 68.741 | 7.5121 | 0.0027 |
| P00747 | Plasminogen | 90.568 | 13.4998 | 0.0029 |
| Q59FR8 | LGALS3 protein variant (Fragment) | 27.117 | 4.3434 | 0.0030 |
| P36952 | Serpin B5 | 42.1 | 9.0944 | 0.0031 |
| P06727 | Apolipoprotein A-IV | 45.398 | 27.1755 | 0.0039 |
| E7EX29 | 14-3-3 protein zeta/delta (Fragment) | 28.036 | 12.1152 | 0.0070 |
| P00558 | Phosphoglycerate kinase 1 | 44.614 | 16.8734 | 0.0080 |
| P04792 | Heat shock protein beta-1 | 22.782 | 11.3353 | 0.0080 |
| Q01518 | Adenylyl cyclase-associated protein 1 | 51.901 | 9.0202 | 0.0090 |
| P00338 | L-lactate dehydrogenase A chain | 36.688 | 30.5144 | 0.0094 |
| P19827 | Inter-alpha-trypsin inhibitor heavy chain H1 | 101.39 | 40.5268 | 0.0098 |
| P04004 | Vitronectin | 54.305 | 17.1521 | 0.0106 |
| Q14508 | WAP four-disulfide core domain protein 2 | 12.993 | 4.1482 | 0.0151 |
| Q6PIQ7 | IGL@ protein | 25.021 | 10.3262 | 0.0168 |
| P15311 | Ezrin | 69.412 | 11.0967 | 0.0204 |
| P31949 | Protein S100-A11 | 11.74 | 4.7898 | 0.0207 |
| J3KPS3 | Fructose-bisphosphate aldolase | 39.817 | 11.7024 | 0.0209 |
| P13796 | Plastin-2 | 70.288 | 8.9283 | 0.0226 |
| E9PK25 | Cofilin-1 | 22.728 | 14.1857 | 0.0256 |
| P35321 | Cornifin-A | 9.8774 | 16.2797 | 0.0267 |
| O00151 | PDZ and LIM domain protein 1 | 36.071 | 5.0495 | 0.0296 |
| Q04828 | Aldo-keto reductase family 1 member C1 | 36.788 | 15.0607 | 0.0339 |
| B7ZKJ8 | 35 kDa inter-alpha-trypsin inhibitor heavy chain H4 | 103.88 | 17.3192 | 0.0387 |
| E7EMB3 | Calmodulin | 21.689 | 14.3018 | 0.0454 |
| P04083 | Annexin A1 | 38.714 | 6.4440 | 0.0497 |
| P80303 | Nucleobindin-2 | 50.195 | 0.2171 | 0.0004 |
| Q9UII7 | E-cadherin | 99.692 | 0.2030 | 0.0005 |
| P02671 | Fibrinogen alpha chain | 94.972 | 0.1814 | 0.0129 |
| Q6UXB2 | VEGF co-regulated chemokine 1 | 13.819 | 0.1410 | 0.0336 |
