# Supplementary material for: Characterization of human tear proteome reveals differentially abundance proteins in thyroid-associated ophthalmopathy
Source: PeerJ. 2022 Jul 12;10:e13701. doi: 10.7717/peerj.13701 (PMC9285480; doi:10.7717/peerj.13701)
Supplement: Supplemental Information 4 [file peerj-10-13701-s004.docx]

Subject's original information sheet

|  | TAO group | | HC group | |
| --- | --- | --- | --- | --- |
| Sample | Age | Sex | Age | Sex |
| 1 | 34 | F | 50 | F |
| 2 | 29 | F | 49 | F |
| 3 | 28 | F | 26 | F |
| 4 | 47 | F | 29 | F |
| 5 | 51 | F | 44 | F |
| 6 | 41 | F | 37 | F |
| 7 | 48 | F | 60 | F |
| 8 | 47 | F | 39 | F |
| 9 | 30 | F | 37 | F |
| 10 | 29 | F | 25 | F |
| 11 | 52 | F | 36 | F |
| 12 | 28 | F | 30 | F |
| 13 | 38 | F | 35 | F |
| 14 | 32 | F | 22 | F |
| 15 | 30 | F | 45 | F |
| 16 | 35 | M | 35 | M |
| 17 | 48 | M | 31 | M |
| 18 | 53 | M | 25 | M |
| 19 | 44 | M | 40 | M |
| 20 | 39 | M | 48 | M |
| 21 | 48 | M | 55 | M |
| 22 | 42 | M | 43 | M |
| 23 | 31 | M | 28 | M |
| 24 | 36 | M | 34 | M |
| 25 | 51 | M | 25 | M |
| 26 | 28 | M | 27 | M |
| 27 | 46 | M | 42 | M |
| 28 | 42 | M | 31 | M |
| 29 | 35 | M | 46 | M |
| 30 | 35 | M | 52 | M |
